# Power structure in Chilean news media

**DOI:** 10.1371/journal.pone.0197150

**Published:** 2018-06-06

**Authors:** Jorge Bahamonde, Johan Bollen, Erick Elejalde, Leo Ferres, Barbara Poblete

**Affiliations:** 1 Department of Computer Science, University of Chile, Santiago, Chile; 2 School of Informatics and Computing, Indiana University, Bloomington, IN, United States of America; 3 Computer Science Department, Faculty of Engineering, Universidad de Concepción, Concepción, Chile; 4 Institute of Data Science, Faculty of Engineering, Universidad del Desarrollo, Santiago, Chile; 5 Telefónica R&D, Santiago, Chile; Consejo Nacional de Investigaciones Cientificas y Tecnicas, ARGENTINA

## Abstract

Even democracies endowed with the most active free press struggle to maintain diversity of news coverage. Consolidation and market forces may cause only a few dominant players to control the news cycle. Editorial policies may be biased by corporate ownership relations, narrowing news coverage and focus. To an increasing degree this problem also applies to social media news distribution, since it is subject to the same socio-economic drivers. To study the effects of consolidation and ownership on news diversity, we model the diversity of Chilean coverage on the basis of ownership records and social media data. We create similarity networks of news outlets on the basis of their ownership and the topics they cover. We then examine the relationships between the topology of ownership networks and content similarity to characterize how ownership affects news coverage. A network analysis reveals that Chilean media is highly concentrated both in terms of ownership as well as in terms of topics covered. Our method can be used to determine which groups of outlets and ownership exert the greatest influence on news coverage.

## Introduction

Chomsky once commented on the role of a free and diverse press: “The smart way to keep people passive and obedient is to strictly limit the spectrum of acceptable opinion, but allow very lively debate within that spectrum.” [1] This is in fact the position that many advanced democracies find themselves in as the diversity of news coverage seems to shrink, whereas news coverage itself seems to continuously expand in a non-stop news cycle. For example, the US has gone from 50 companies in 1983 to only 6 companies that control 90% of media outlets in 2000, with further consolidation possible in the future [2]. Lack of diversity of viewpoints, topics, and representation of communities has been attributed to this relentless process of consolidation [3].

The emerging lack of diversity and coverage in news reporting is frequently attributed to two specific factors. First, as news media outlets attempt to cater to their audiences or community, they narrow their coverage to community-specific material, and as a consequence further limit their audience awareness in a homophilic cycle of mutual preferential attachment. This effect has recently been studied in terms of so-called online “filter bubbles” [4], in which users can choose to subscribe to outlets and news that confirm their pre-existing preferences and view-points, not only narrowing their own media exposure, but also encouraging outlets to increasingly specialize to smaller and more defined communities [5].

Second, the market-driven consolidation of the news media industry may lead to concentration of ownership. According to Chomsky’s Propaganda model [6], this concentration of ownership may have direct and indirect effects on editorial policies. For example, the editorial board of a newspaper owned by a group that also invests in agriculture may perceive a pressure to report more favorably about agricultural initiatives. Unlike other factors such as the (frequently explicitly publicized) political and historical mission of the outlet and its readership, ownership bias may thus exert a more insidious effect on editorial policies that is difficult to operationalize and quantify. Nevertheless, it may have a significant effect on the degree to which news consumers perceive the world and their ability to gather objective and effective information.

With the emergence of online social media platforms, most news outlets, from the smallest to the largest, have established an online presence that they use for real-time distribution of news content [7]. These online environments provide an opportunity to test hypotheses with respect to the drivers affecting news diversity, such as consolidation, coverage, ownership, and network homophily.

Twitter [8] is a prime example of a social media platform geared towards the real-time distribution of news. Twitter enables its users to post short messages, called “tweets”, that can be up to a maximum of 140 characters in length. Users can choose to subscribe to the tweets posted by other users. Once they “follow” a given user they will receive that user’s Tweets in their own feed. Tweets can originate from individual users, but also from news outlets and other organizations. In fact, the large majority of news outlets post their most recent headlines and a brief summary of their news on their Twitter accounts in real-time. They are followed by large numbers of Twitter users who have made Twitter their primary news source.

The Twitter platform is particularly interesting as foundation for the study of news diversity and coverage since it is designed to constitute a large-scale social network. The ensemble of users-following-users establishes a social network where tweets travel along the edges of the network. This renders the social media platform an ideal laboratory to apply the toolkit of network science to the investigation of news diversity and coverage from a top-down (user to user to news outlet) as well as a bottom-up perspective (news outlets to their followers).

In our current study we research the influence that ownership relations have on news media content and coverage by quantifying the strength of the relation between news media ownership and news media content diversity in Twitter. We analyze the user accounts of news media outlets to study how their content evolves and overlaps, and whether or not these observations are linked to their known ownership structure.

We focus on Chilean news outlets since they have established a significant social media presence with a high number of Chilean users per 1000 individuals [9]. In addition, Chilean news has a clearly defined national audience which is geographically and culturally well-demarcated, thereby providing a distinct sample from previous media studies that were focused on English-speaking countries. The Chilean media landscape is furthermore well documented due to the availability of detailed, publicly available data with respect to its ownership structure, compiled by *Poderopedia*, a journalist NGO that aims to understand power relationships between people, companies, and organizations.

We use the latter information to trace the existing ownership structure of Chilean media outlets which we then compare to the structural properties of their Twitter coverage and content, in particular with respect to the similarity of the content they publish in social media. To this end, we define a series of different content similarity metrics and evaluate for each one its relation to ownership.

Prior work has focused on studying story selection similarity within different news outlets [10]. However, we extend this research by searching for indications of deeper interconnections in the *mediasphere* at the intra-country level. An *et al.* [11] modeled the outline of digital media on Twitter, analyzing media similarity based on the degree of overlap between their respective follower communities. They reported a strong tendency for members of the communities to read news from multiple sources, mostly on similar topics. Kang *et al.* [12] proposed an approach for capturing unique and newsworthy topics from Twitter, which have not been published by mainstream media. They studied ways to measure the correspondence between short-text messages and news media articles using content similarity and network information. Park *et al.* [13] proposed a system to identify and track events, in order to present different points of view of the same affair to readers to counteract opinion bias in news.

In relation to news bias analysis, Gilens & Hertzman [14] performed a case study of how newspapers covered the 1996 Telecommunications Act in the U.S. In particular, they observed that media outlet ownership, and their relation to the proposed regulation change, had a direct influence in the coverage of that information. In a similar subject, Saez-Trumpe *et al.* [15] defined a methodology to identify “selection” or “gatekeeping”-bias, which consist of editorial decisions to publish certain stories and not others. They study these biases with respect to the prominence of the stories and the geographical location of the outlet. Since their work uses a data set of media from different countries, they find that geography might influence the selection of the stories. Zhao *et al.* [16] studied the coverage of news on Twitter in relation to traditional news media using topic models. They observe similar topic coverage, but with a different distribution. They found that Twitter users write less about world events, however they are quick to forward (re-post) these news.

Our work complements prior research by searching for potential causal pathways to explain the homophilic relations between groups of news outlets. This might help to identify and characterize potential filter bubbles, possibly informing novel recommendation system aimed at increasing the diversity of news consumption.

## Materials and methods

Our goal is to analyze whether ownership and content are correlated in the domain of digital media news outlets. We approach this problem by studying the similarity networks and clusters that emerge from the content published on Twitter by news outlets in Chile. We contrast groups of similar news accounts with their ownership in the real-world to see if they are related according to different similarity metrics.

In particular, we study the similarity between pairs of news accounts from several perspectives: *vocabulary*, *keyword-based topics*, and *minhash-based topics*. We aim to determine if there exist consistent similarity-based communities among news media outlets and if this same consistency arises in relation to ownership.

In order to achieve this, we perform independent static analyses of news media outlets for two years, 2015 and 2016. For each year, we study the communities of news outlets that are produced by using community detection over similarity graphs built for each similarity metric. In addition, we identify clusters of similar outlets with the purpose of checking consistency of the resulting similarity groups. Below, we detail our similarity metrics, and community and clustering algorithms. Our data analysis was based on the following data sets (we complied with the terms of service for the websites from which we collected data: the Poderopedia database is freely available, and the Twitter data was accessed using their public API):

- **Chilean News Twitter (*ds15*):** This data set was created by Maldonado *et al.* [17] for their study that characterized Chilean news events. It consists of a collection of 714,973 tweets, which were posted by 84 prominent Chilean news media outlets, from October 30th, 2014 through May 20th, 2015 (including retweets).
- **Chilean News Twitter (*ds16*):** This data set consists of a collection of 756,864 tweets, which were posted by 365 Chilean news media outlets from October 25, 2015 to January 25, 2016 (including retweets). This collection was based on an exhaustive and manually curated list of news outlets, derived from the Wikipedia page listing Chilean news media [18] and the independent journalistic website Poderopedia.

Both data sets were obtained by downloading all of the tweets posted by each news outlet during the time period of each collection. Tweet metadata was kept for each data set, such as location and user identifiers. In addition, we performed standard text pre-processing of the tweet content. This consisted of term tokenization on white-space boundaries, conversion to lower-case, and the removal of stop-words, URLs, punctuation marks and other non-alphanumeric characters. In addition, we removed news outlets that posted less than one tweet per day on average, which left 79 news outlets in *ds15* and 341 in *ds16*.

As for ownership information, we manually mapped Poderopedia’s influence database [19] to our lists of news media accounts on Twitter. As for grouping news media outlets according to their owners, we simply consider two outlets to belong to the same group if and only if they’re owned by the same entity. There are at least two possible issues with this. First, some news media outlets are owned by multiple entities: in this case, we selected the major partner. On the other hand, there also exist ownership relationships *between* owners. In this case, we selected the entity that subsumes all others as the owner. As a result we obtained the first complete database of newspaper ownership information in Chile (see https://github.com/eelejalde/Chilean_Media_Power_Structure).

Our datasets include news outlets that belong to the two biggest news media groups in Chile: the *El Mercurio* group and the *Copesa* media conglomerate, which form what has been called in the past a newspaper duopoly [20]. We also have representatives of a group of digital newspapers, the *Mi Voz* network. Other owners with smaller number of outlets are also included, as well as a group of *unknown-to-us ownership*. We note that we are interested not only in news outlets that share owners, but also those that behave as if they did.

### Similarity metrics between news outlets

We are interested in finding how related news outlets are using their content. In other words, we study their vocabularies and how the stories they select may indicate a connection between their editorial policy. For topic-based similarity metrics, we decided to use keyword- and minhash-based similarity to validate the consistency of the results beyond the selected methodology.

1. **Vocabulary-based similarity.** We model each news media outlet as a single document composed of all of the tweets posted by its news account during the time of our data collection. Each document is converted to its vector-space representation using a *tf-idf* weighting scheme [21]. Similarity is then computed as the cosine similarity between two vectors [22].
2. **Keyword-based topic similarity.** This is a more elaborate notion of content-based similarity between news media outlets, which is based on whether two sources effectively talk about the same topics.
  We identify the topics that were discussed during each day in our dataset. Each topic is obtained by mining frequent term-sets from the tweets posted that day and then joining these sets by word co-occurrence (within the same day).
  A daily vector representation is computed for each outlet based on the day’s topics; daily similarities between pairs of outlets are obtained as the cosine similarities of pairs of these vectors. Finally, the overall topic similarity between two outlets is defined as their average daily similarity.
3. **Minhash-based topic similarity.** This is an alternative similarity measure based on text, inspired by prior work for identifying similar documents. We represent the text of each tweet posted by a news outlet by its *k*-shingles, using word-based shingles and *k* = 3 (this is based on prior findings that indicate that *k* equal to 2 or 3 word shingles are appropriate for short documents [23–25]). We set *k* = 3 to obtain a fine grain classifier identifying specific stories, rather than broader topics for which a smaller value of *k* may have been chosen.
  A 4-min-wise hashing is applied to each tweet representation, this results in compact summaries of documents that are effective for identifying similarity [23, 24, 26]. Accordingly, we cluster tweets using their minhash similarity. We refer to these clusters as *topics*, as this is a notion that has been used in past literature to identify “stories” that relate to a common event or topic among news outlets [10, 15]. Similarity between two news outlets is then defined by the co-occurrence of two news outlets with respect to a same topic. In particular, the value of the similarity between outlet *A* and *B* is the conditional probability *Pr*(*A*|*B*) of the occurrence of *A* in a cluster given that *B* occurs in that same cluster. This similarity measure is directional, expressing how likely it is that a story tweeted by *B* is also tweeted by *A* [11]. In order to define a symmetric similarity measure we further specify the similarity between *A* and *B* as *sim*(*A*, *B*) = *max*(*Pr*(*A*|*B*), *Pr*(*B*|*A*)).

### Similar news outlet identification

In order to identify similar news outlets, we create different *similarity graphs* based on each of the aforementioned similarity metrics and each of the datasets. Formally, we define a generic similarity graph *G* = (*V*, *S*) for the set of news outlets *V* = {*v*_1_, *v*_2_, …, *v*_*n*_} and similarity measure *S*: (*v*_*i*_, *v*_*j*_) → IR^+^, as a graph where each pair of outlets *v*_*i*_ and *v*_*j*_ are connected by an edge of weight *S*: (*v*_*i*_, *v*_*j*_). This yields a complete, weighted, and undirected graph.

Using each similarity graph, we apply graph partitioning techniques to find groups of similar news outlets. For all six similarity graphs we used a hierarchical, agglomerative community discovery algorithm [27], and the normalized cut technique [28]. This methodology has been proved to be successful in similar problems [29].

## Results and discussion

**Fig 1:**
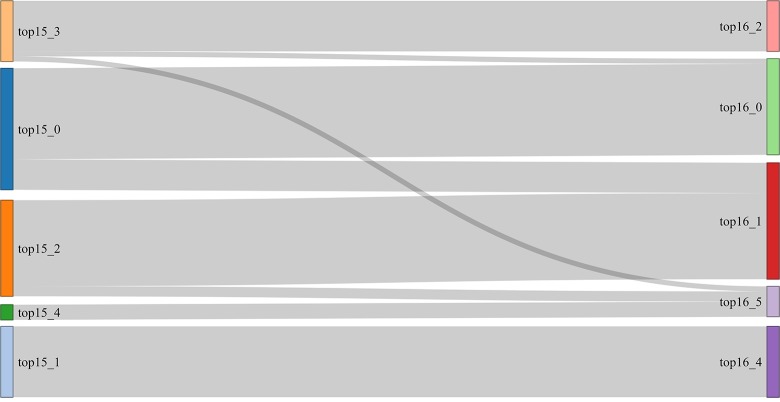
Similarity of *ds15* vs. *ds16* communities on *topic (keyword-based)*.

**Fig 2:**
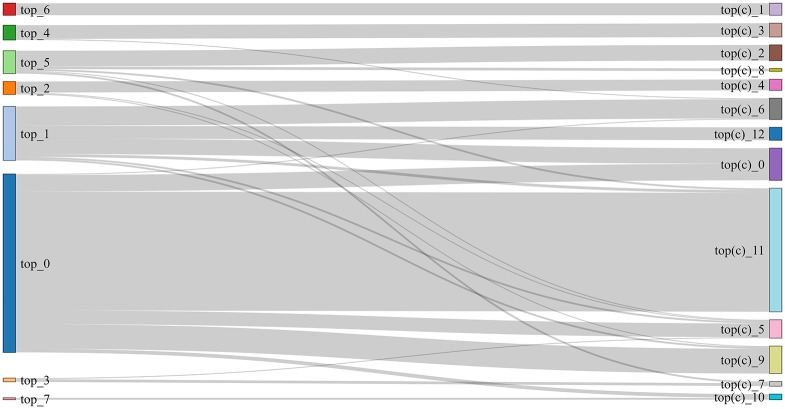
Similarity of communities vs. clusters resulting from *ds16* on *topic (keyword-based)*.

As Fig 1 shows, the communities (represented as boxes) of news outlets obtained from Topics collected in *ds15* (left column) and *ds16* (right column). The curves that connect both columns of boxes represent the number of outlets (or the proportion of outlets) shared by the communities at the two ends of the curve. For example, the first community in the top left (from *ds15*) has 18 (3/4) outlets in common with the first community in the top right (from *ds16*) and shares only 6 (1/4) outlets with the second. Also, the last community in left is identical to the last community in the right. Since results do not vary significantly between the two datasets, and to save space, we only report results for one of the datasets; the more recent one, *ds16*. In the same vein, we only report results for one of the graph partitioning algorithms (community detection) since the resulting communities in both cases are very similar. This congruence supports the notion that the communities found are significant, denoting a real structure in the data (see Fig 2). In any case, the rest of the analysis can be found online at https://github.com/eelejalde/Chilean_Media_Power_Structure.git and in S1–S8 Tables. This notwithstanding, there were some cases for which differences were found, and we do report these, making it clear where they come from.

Table 1 summarizes several metrics for community discovery over both data sets. The column *Outlets* is the initial number of outlets in the similarity graph (see Section Materials and methods), while *Grouped* is the number of outlets that were included in one community. Column *Comm.* is the number of communities found by the algorithm, while *Mod.* and *Cond.* show, respectively, the modularity and conductance of the sub-graphs formed by the *Grouped* outlets within returned communities (see Section Materials and methods).

**Table 1:** Internal metrics for community structures derived from each explored similarity measure for the *ds15* and *ds16* datasets.

Similarity	Outlets		Grouped		Comm.		Mod.		Cond.	
	*ds15*	*ds16*	*ds15*	*ds16*	*ds15*	*ds16*	*ds15*	*ds16*	*ds15*	*ds16*
Vocabulary	79	341	52	262	7	14	0.38	0.38	0.02	0.35
Topics	79	341	50	133	4	7	0.60	0.58	0.11	0.26
MinHash	75	365	50	355	6	11	0.40	0.74	0.01	0.04

Grouped outlets (Grouped) correspond to those belonging to a discovered community. Modularity (Mod.) and conductance (Cond.) are calculated with respect to this subgraph.

The first thing to notice is that *Topic* similarity creates the lowest number of communities. Also, this similarity for the Dataset *ds15* includes almost all outlets, but for *ds16* it only grouped about a third of the total dataset. We think this is because this similarity is a coarse-grained classification that only captures the strongest signals. If we focus on *ds15*, the *topic* similarity has the highest modularity, which means it creates well defined communities. However, we have to take into account that this dataset only contains 84 outlets that comprise most of the largest, most famous newspapers of the country. When we look at the *ds16* dataset we find a more diverse set of outlets (in size and content). In *ds16*, *Topic* similarity shows similar performance if un-grouped outlets are excluded. In turn, *MinHash* seems to be more sensitive to weaker signals, creating a more fine-grained classification. We can see this in the high modularity achieved with *ds16* in spite of having included most outlets. On the other hand, *Vocabulary* similarity has the lowest performance in both datasets, which gives us the intuition that there are no particularly strong differences in vocabulary between the analyzed outlets.

### Vocabulary similarity

Communities obtained for the *Vocabulary* similarity over the *ds16* dataset include four big communities and ten smaller ones. Most of the smaller communities form around geographical information, as their relevant words include city names and places. In some cases, the outlet names also denote a geographical region, as many regional outlets have location-related names: “El Mercurio de Valparaíso”, for example, is based in the city of Valparaíso. Thus, for some communities, we can infer regional-focused content by manually inspecting the names of the outlets grouped within it. We also find small communities pointing to a particular topic, such as aquaculture, and a particular type of media, such as radio stations. This is possibly caused by the fact that these two characteristics heavily influence the respective outlets’ vocabularies: aquaculture is a very specific activity with its own terms, and radio stations tend to use a more informal language than other media outlets (see S1 Table for more detail).

There is also a pair of big communities that seems to comprise many different outlets: the first has many national-scope media, while the other has many that seem oriented toward regional locations. The characterization of ownership for the same set of communities can be seen in Table 2. Notice that over half of the community with ID 4 is owned by El Mercurio. Similarly, over half of the community with ID 6 is owned by Grupo Diarios en Red. On the other hand, the other two big communities (IDs 2 and 7) do not have any owner that heavily dominates the group. For the rest of the communities, even when they are very small ones, it is difficult to find homogeneity in the ownership of the groups. These results suggest a low influence of ownership of a news outlet over the vocabulary they use. The clustering algorithm over this similarity graph confirms the results with a similar distribution of ownership over the clusters (see S2 and S3 Tables).

**Table 2:** Ownership properties for vocabulary-based communities for the *ds16* dataset.

ID	Size	Main owner(s)	Owner(s)% [#]	Unk. owner% [#]
0	79	Copesa	6.33 [5]	25.32 [20]
1	3	Red de Diarios Comunales	66.67 [2]	0.00 [0]
2	91	Copesa	14.29 [13]	4.40 [4]
3	4	Empresa Periodistica y Radiodifusora Las Nieves	25.00 [1]	0.00 [0]
		Sociedad Periodistica de Aysen	25.00 [1]	
		Sociedad Editora y Periodistica La Verdad	25.00 [1]	
		Sociedad El Patagon Domingo	25.00 [1]	
4	43	El Mercurio	55.81 [24]	11.63 [5]
5	2	Comunicaciones Mia	50.00 [1]	0.00 [0]
		Grupo Prisa	50.00 [1]	
6	23	Grupo Diarios en Red	56.52 [13]	17.39 [4]
		El Mercurio	17.39 [4]	
7	78	Asesorias e Inversiones Comunidades Ciudadanas	14.10 [11]	29.49 [23]
		El Mercurio	11.54 [9]	
8	2	Mono Manco	50.00 [1]	0.00 [0]
		Camilo Montalban Araneda	50.00 [1]	
9	6	Sociedad Periodistica e Impresora el Labrador	16.67 [1]	33.33 [2]
		Portal de Melipilla	16.67 [1]	
		Editora el Centro Empresa Periodistica	16.67 [1]	
		Antonio Puga	16.67 [1]	
10	2	-	-	100.00 [2]
11	2	Red de Diarios Comunales	50.00 [1]	0.00 [0]
		Sociedad Periodistica Banic y Lancelloti	50.00 [1]	
12	2	Tu Ciudad Virtual	50.00 [1]	50.00 [1]
13	2	Editec	50.00 [1]	0.00 [0]
		Sociedad Medios Comunicaciones	50.00 [1]	
14	2	Grupo Prisa	100.00 [2]	0.00 [0]

The community with an ID of 0 corresponds to un-grouped media outlets. Entities owning over 10% of the outlets in a community are listed next to it.

### Topic similarity: Keyword based

The computed community structure has a big community containing many national and local-scope media outlets, whose most relevant keywords are mainly centered around political and sports figures and current issues. A small community is particularly oriented towards one particular region, Valparaíso; analogously, another community seems to be focused on another region, Aconcagua Province (see S4 Table for more detail). The remaining communities seem to lack a unifying theme, displaying topics related to general advice, geographical entities and buzzwords that aim to capture audience interest.

When ownership comes into play, these remaining communities acquire meaning (see Table 3). The communities with IDs 2, 4, 5 and 6 have each an entity owning over 80% of them. In contrast to the results seen before for the *Vocabulary* similarity, this indicates that ownership might have an influence on topics discussed.

**Table 3:** Ownership properties for keyword-based communities for the *ds16* dataset.

ID	Size	Main owner(s)	Owner(s)% [#]	Unk. owner% [#]
0	208	Grupo Copesa	5.29 [11]	25.96 [54]
1	59	Copesa	11.86 [7]	6.78 [4]
2	14	El Mercurio	85.71 [12]	14.29 [2]
3	4	Medios de Consorcio Periodistico El Epicentro	25.00 [1]	0.00 [0]
		Corporacion de Television de la Pontificia Universidad Catolica de Valparaiso	25.00 [1]	
		Comunicaciones Pacifico	25.00 [1]	
		Radio Festival	25.00 [1]	
4	16	Asesorias e Inversiones Comunidades Ciudadanas	93.75 [15]	0.00 [0]
5	25	El Mercurio	96.00 [24]	0.00 [0]
6	13	Grupo Diarios en Red	100.00 [13]	0.00 [0]
7	2	Patricio Gallardo Montenegro	50.00 [1]	50.00 [1]

The community with an ID of 0 corresponds to un-grouped media outlets. Entities owning over 10% of the outlets in a community are listed next to it.

Outlets owned by groups like *El Mercurio* or *Diarios en Red* have recognizable clusters using the agglomerative community detection algorithm (also shown with the normalized cut clustering in S5 and S6 Tables). The *Copesa* group (the closest competitor of *El Mercurio*) does not have its own, clearly defined, community. This may be due to the kind of outlets it owns (a lot of them are magazines specialized in different topics). This makes it harder to identify any owner influence on their editorial strategies, which seems to be a limitation of the current methodology.

### Topic similarity: Minhash-based

Using the *minhash* technique over the tweets in *ds16*, we identified 100,774 topics that contain 438,353 tweets. In the case of *ds15*, we identify 83,582 topics containing a total of 254,650 tweets. We looked for topics that had tweets from multiple news outlets. In *ds16*, out of all the topics, 31,423 contained tweets from more than one news outlet (31.2%) and in *ds15* 17,211 (20.6%). Using these topics, we used the co-occurrences for each pair of news outlets to calculate their similarity (see Section Materials and methods). We found that all news outlets co-occur at least once with some other news source, for both *ds16* and *ds15*.

**Fig 3:**
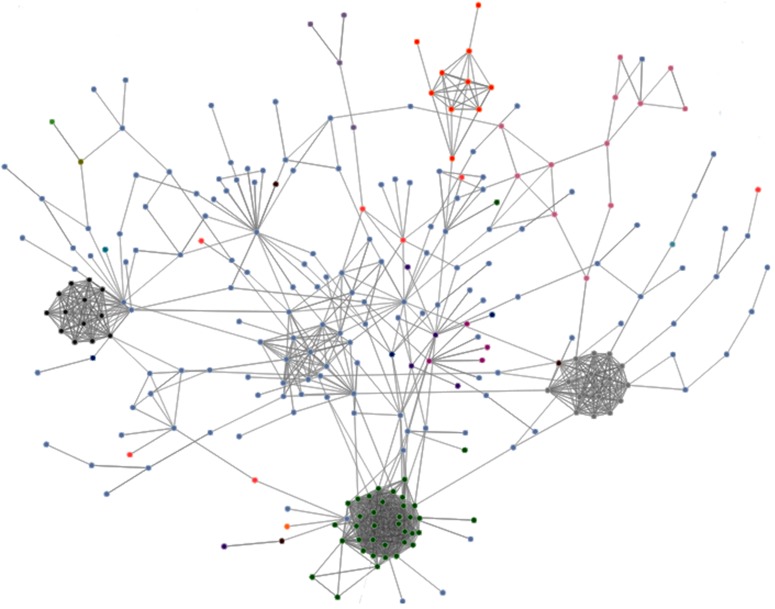
Similarity graph, using *topic (MinHash-based)* similarity on *ds16*. Only representing edges with weight over (mean+2std). We assigned different colors to the biggest owners.

Results for Minhash-based similarity have features like those seen in the keyword-based communities. These communities are easily identifiable even by visual inspection (see Fig 3). We observe two big communities with many different media outlets (with IDs 1 and 3), some small ones (with IDs of 4, 5, 10 and 11) which, as before, under manual inspection of the outlet names seem to have specific scopes (*e.g.* the Linares Province, aquaculture, the Chiloe Province or the Maipú commune). S7 Table shows a more detailed view of the communities.

Ownership features help explain the remaining communities as shown in Table 4. Communities 2, 6, 7 and 9 have entities that own a big part of them. The small community with an ID of 11 does not have a clear meaning or unifying theme. Notice that in this case the community with ID of 3 has similar characteristics to the communities with ID 0 for the other similarity measures (the un-grouped outlets). This community has the biggest number of outlets and the main owner has less than 10% of them.

**Table 4:** Ownership properties for minhash-based communities for the *ds16* dataset.

ID	Size	Main owner(s)	Owner(s)% [#]	Unk. owner% [#]
0	10	El Mercurio	20.00 [2]	30.00 [3]
1	109	Red de Diarios Comunales	12.84 [14]	21.10 [23]
2	43	El Mercurio	83.72 [36]	11.63 [5]
3	130	Copesa	4.62 [6]	30.00 [39]
4	2	Radio Ancoa de Linares	50.00 [1]	0.00 [0]
		Comunicaciones del Sur	50.00 [1]	
5	2	Editec	50.00 [1]	0.00 [0]
		Sociedad Medios Comunicaciones	50.00 [1]	
6	9	Betazeta Networks	100.0 [9]	0.00 [0]
7	36	Asesorias e Inversiones Comunidades Ciudadanas	41.67 [15]	30.56 [10]
8	6	El Mercurio	16.67 [1]	33.33 [2]
		Copesa	16.67 [1]	
		Troya Comunicaciones	16.67 [1]	
		Servicios de Radio Difusion Pedro Felidor Roa Barrientos	16.67 [1]	
9	14	Grupo Diarios en Red	92.86 [13]	0.00 [0]
10	2	Mono Manco	50.00 [1]	0.00 [0]
		Camilo Montalban Araneda	50.00 [1]	
11	2	Sociedad Radiodifusora Primordial FM	50.00 [1]	50.00 [1]

The community with an ID of 0 corresponds to un-grouped media outlets. Entities owning over 10% of the outlets in a community are listed next to it.

Even though the communities in *Minhash-based* similarity graph are partially explained by ownership, the correlation is not as strong as in the clustering obtained from the normalized cut algorithm. As mentioned above, this is one case where we think the differences between the two algorithms are worth mentioning: for this particular similarity graph the normalized cut clustering algorithm actually improves results (see Table 5)(see also S8 Table).

**Table 5:** Ownership properties for minhash-based clustering for the *ds16* dataset.

ID	Size	Main owner(s)	Owner(s)% [#]	Unk. owner% [#]
0	245	Copesa	5.71 [14]	25.71 [63]
1	14	-	-	50.00 [7]
2	19	El Mercurio	100.0 [19]	0.00 [0]
3	16	Asesorias e Inversiones Comunidades Ciudadanas	93.75 [15]	0.00 [0]
4	14	El Mercurio	100.0 [14]	0.00 [0]
5	13	Grupo Diarios en Red	100.0 [13]	0.00 [0]
6	9	Grupo Prisa	100.0 [9]	0.00 [0]
7	4	Editorial Televisa Chile	100.0 [4]	0.00 [0]
8	1	Betazeta Networks	100.0 [1]	0.00 [0]
9	14	Red de Diarios Comunales	85.71 [12]	0.00 [0]
10	3	Estado de Chile	33.33 [1]	0.00 [0]
		ITV Patagonia	33.33 [1]	
		Corporacion de Television de la Pontificia Universidad Catolica de Valparaiso	33.33 [1]	

Entities owning over 10% of the outlets in a cluster are listed next to it.

If we assume that the biggest cluster (with ID 0) is the one containing the outlets that do not fit in any other group (equivalent to the un-grouped outlets in the community detection), then we get clusters that are very similar to the communities we obtained for *topic (keyword-based)* similarity.

On one hand, the clusters leave out a bigger number of outlets than the community structure. This reduces the number of clustered outlets to an amount similar to that found with *topic (keyword-based)* similarity. On the other hand, it finds a classification with a better owner separation. As we can see in Table 5, there are two relatively small clusters (with ID 8 and 10). Beside those two, all other clusters are heavily, if not entirely, dominated by one owner.

### Clustering metrics

Based on these results, we hypothesize that ownership relationships are similar to the ones based on content. Given that we have the actual owners of most news outlets in our data sets, we used this as a ground truth to evaluate the performance of our methodology. To this end, we computed different clustering metrics using ownership information as class labels [30, 31].

We used the Adjusted Rand Index (**ARI**) to quantify the degree of correspondence between the set of communities found by our methodology and the sets of clusters defined by the actual owners of the news outlets. **ARI** scores are normalized against chance, so scores close to 0.0 indicate random label assignments, 1.0 indicates a perfect match, and negative scores indicate a correspondence lower than what is expected for random assignments. Similarly, the Adjusted Mutual Information (**AMI**) index gives a sense of how much information we can obtain about one distribution given the other one. **AMI** scores are also adjusted with respect to the expected value (subtracting the expected value from the Mutual Information score). Again, scores close to 0.0 indicate random assignments and a 1.0 score indicates two identical assignments. The Normalized variation of the Mutual Information Index (**NMI**) also gives a greater score as the communities are closer to a perfect recreation of ownership classes. Moreover, **NMI** does not penalize if the classes are further subdivided into smaller clusters. The results of the application of these indices (given in Table 6) suggest non-random clusters. Homogeneity (**Hom**) is maximized when each cluster contains members of a single class, while completeness (**Com**) measures the desirable objective of assigning all members of a class to a single cluster.

**Table 6:** Comparison of community structures and ownership.

Similarity	Outlets	Comm.	ARI	AMI	NMI	Hom	Com
Vocabulary	157	28	0.1834	0.3007	0.5362	0.4748	0.6056
Topic: Keywords	157	66	0.4246	0.4261	0.7313	0.8113	0.6592
Topic: Minhash	167	12	0.4301	0.4584	0.6593	0.5460	**0.7961**
Cluster Topic: Minhash	169	90	**0.5326**	**0.4652**	**0.8365**	**1.0000**	0.6997

Rows represent the different metrics used to calculate the similarity graphs. Columns represent the scores of the indices calculated using our ground-truth as reference (**ARI**: Adjusted Rand Index, **AMI**: Adjusted Mutual Information Based, **NMI**: Normalized Mutual Information Based, **Hom**: Homogeneity, **Com**: Completeness)

Prior to calculations, we removed outlets without ownership information and outlets with owners that only have a single outlet, since they do not add any relevant information. Additionally, there are outlets that do not belong to any of the communities we found. They could be discarded, but we might be deleting valuable information: our algorithm indicates their content is different from the others’. For this reason, we preserve each of them as a community of size 1. Though reasonable, this might distort some comparison metrics, as the correspondence of single-outlet communities is perfect if they’re isolated in both the content-based and the owner-based community structures. As both the number of considered outlets and the number of communities is altered by these decisions, we specify them in Table 6 (columns *Outlets* and *Comm.* respectively).

**Fig 4:**
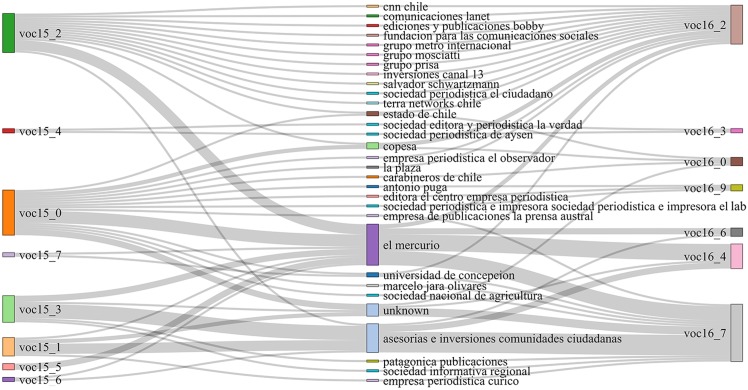
Ownership vs. vocabulary community structure. Owners are displayed on the center, while communities are displayed on the left for ***ds15***, and on the right for ***ds16***. The width of a flow connecting an owner and a community is proportional to the number of outlets in the community belonging to that owner.

**Fig 5:**
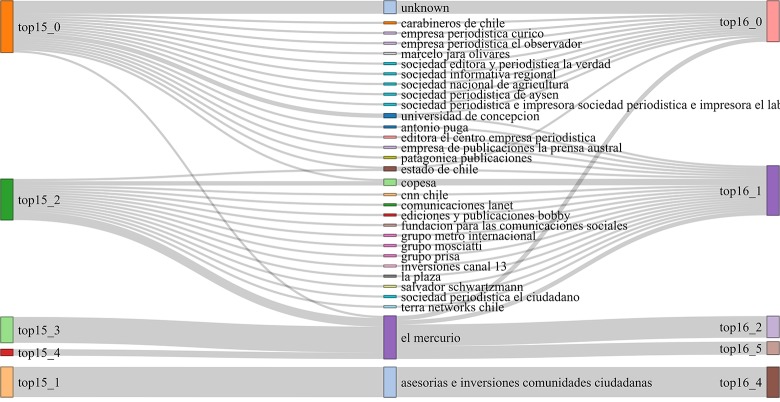
Ownership vs. topic (keyword-based) community structure. Owners are displayed on the center, while communities are displayed on the left for ***ds15***, and on the right for ***ds16***. The width of a flow connecting an owner and a community is proportional to the number of outlets in the community belonging to that owner.

**Fig 6:**
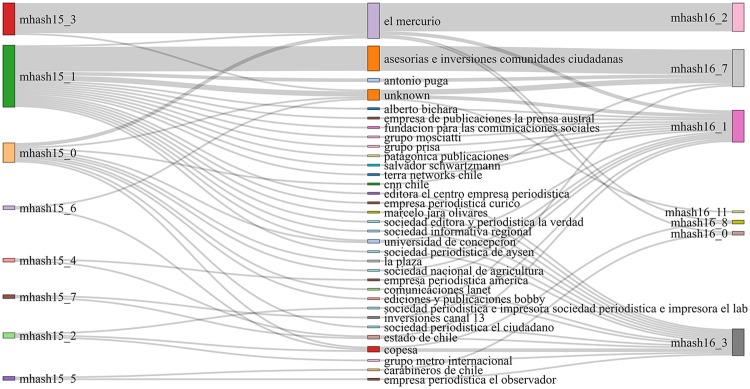
Ownership vs. topic (minhash-based) community structure. Owners are displayed on the center, while communities are displayed on the left for ***ds15***, and on the right for ***ds16***. The width of a flow connecting an owner and a community is proportional to the number of outlets in the community belonging to that owner.

**Fig 7:**
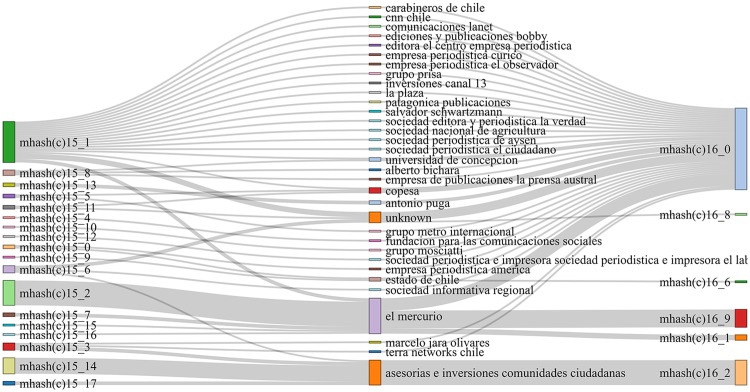
Ownership vs. topic (minhash-based) clustering structure. Owners are displayed on the center, while clusters are displayed on the left for ***ds15***, and on the right for ***ds16***. The width of a flow connecting an owner and a community is proportional to the number of outlets in the community belonging to that owner.

Table 6 shows the results of these indices over the communities obtained from our similarity graphs. Once again, we can see that the vocabulary-based similarity does not give a good prediction on news outlets that belong to the same owner: *Vocabulary* gets the lowest score for all metrics. This poor behavior is shown in a more graphical way in Fig 4. On the other hand, we can see that topic similarities do show higher degrees of correspondence with owner classes, which is consistent with previous observations. Keyword-based similarity communities have high homogeneity (see Fig 5), while Minhash-based communities show very high completeness, (shown in Figs 6 and 7).

We also included in Table 6 the results for indices over the normalized cut clustering obtained for the *topic (MinHash-based)* similarity. We follow the same procedure for clusters, i.e., we removed outlets without ownership information and outlets with owners that only have a single outlet. Also, we moved each remaining outlet in cluster ID 0 to its own individual cluster. The completeness (**Com**) is altered by the bigger number of clusters of size one created from outlets in the cluster with ID 0. The results for this variation (clusters over minhash-based similarity) are the highest for most indices, presenting this technique as a very good predictor for a common-owner relationship.

## Conclusions

We introduced an analysis of Chilean news media outlets based on the information that each news source chooses to post in Twitter and the similarity clusters of news outlets that arise from this content. The study whether ownership influences the content produced by different news sources is an important area of research to make possible biases explicit.

In general, our results indicate that ownership does play an important role in news content similarity. Big, national-scope media with big audiences tend to group together in their own community; other outlets, generally with a more local scope, group according to a mix of ownership and some geographical features that we inferred (such as *Mercurio de Valparaíso*).

We studied several similarity metrics as well as different ways in which to identify clusters (or communities) in our data. The indices that we calculated (e.g., **ARI** and **AMI**) suggest non-random clusters.

These results seem in agreement with our hypothesis, since similarity based on vocabulary may be attributed to other factors (e.g. geographic zones); on the other hand, our other analyses indicate a correlation between owners and their selection of topics (which can be interpreted as a common editorial policy).

We show that our results are consistent over time. We study two non-overlapping in time datasets, *ds15* and *ds16*, and show that they both present consistent properties.

A limitation of our methodology is that when outlets are too specialized (e.g. magazines on automobiles, fashion, etc.), even if they belong to the same owner, they do not cluster together. This is due to the nature of the stories that they publish, since by design they do not share any significant part of their content. It is therefore difficult to conclude that there is any influence by owners in the content generation of these specific type of outlets.

Some owners only have one news outlet. As we saw in the Results and discussion section, our similarity measures based on topics do a good job in pulling apart these special cases from the biggest groups of owners by clustering them together in a different community (our cluster with *id* 0 above). What we are after, however, is the identification of more than one outlet with a single owner such that content in those different media may be affected by a single editorial line.

We have shown that using a language-independent and fast approach we are able to easily discover “editorial homophily”. This can have several applications, such as help mitigate the “filter bubble effect” in people’s news media consumption by recommending more diverse news sources. It can also help identify “hidden owners”, in the sense that we can identify news sources that behave as if they had a same owner, despite not declaring so publicly. Overall, our findings could help towards promoting a media structure that is less biased towards very few groups.

## Supporting information

S1 Table News outlets for vocabulary-based communities for the *ds16* dataset. The cluster with ID 0 corresponds to un-grouped media outlets.
(PDF)

S2 Table Ownership properties for vocabulary-based clusters for the *ds16* dataset. The cluster with ID 15 corresponds to un-grouped media outlets. Entities owning over 10% of the outlets in a community are listed next to it.
(PDF)

S3 Table Ownership properties for vocabulary clusters for the *ds15* dataset. The cluster with ID 7 corresponds to un-grouped media outlets. Entities owning over 10% of the outlets in a community are listed next to it.
(PDF)

S4 Table News outlets for topic keyword-based communities for the *ds16* dataset. The cluster with ID 0 corresponds to un-grouped media.
(PDF)

S5 Table Ownership properties for topic keyword-based clusters for the *ds16* dataset. The cluster with ID 1 corresponds to un-grouped media outlets. Entities owning over 10% of the outlets in a community are listed next to it.
(PDF)

S6 Table Ownership properties for topic keyword-based clusters for the *ds15* dataset. The cluster with ID 1 corresponds to un-grouped media outlets. Entities owning over 10% of the outlets in a community are listed next to it.
(PDF)

S7 Table News outlets for topic minhash-based communities for the *ds16* dataset. The cluster with ID 0 corresponds to un-grouped media outlets.
(PDF)

S8 Table Ownership properties for topic minhash-based clusters for the *ds15* dataset. The cluster with ID 0 corresponds to un-grouped media outlets. Entities owning over 10% of the outlets in a community are listed next to it.
(PDF)
