# Supplementary material for: Power structure in Chilean news media
Source: PLoS One. 2018 Jun 6;13(6):e0197150. doi: 10.1371/journal.pone.0197150 (PMC5991387; doi:10.1371/journal.pone.0197150)
Supplement: S1 Table — The cluster with ID 0 corresponds to un-grouped media outlets. (PDF) [file pone.0197150.s001.pdf]

S1 Table. News outlets for Vocabulary-based communities for the *ds16* dataset.

Com. ID	Size	Outlets
0	79	diarioelcomunal, sabrosia, elandacollino, primordialfm, radiosolchile, candela_fm, cappissima, realcondorito ...
1	3	elvicunense, elpaihuanino, elquiglobal
2	91	cooperativa, canal_13c, nacioncl, t13, bolido_com, cosmochile, chilebcl, uchileradio, platosycopas, mt_motore ...
3	4	diariodeaysen, radiolasnieves, patagondomingo, ddivisadero
4	43	austral_osorno, radiovalparaiso, elrepuertero, soysanantonio, soytome, radio_festival, ellanquihue ...
5	2	40chileoficial, fmok
6	23	el_timeline, antofagastatv, diarioafta, redarica, diariosenred, red_coquimbo, redantofagasta, soyantofagasta ...
7	78	vallenardigital, radionuble, rsbchile, el_serenense, elrancaguino, austral_losrios, laopinon, ultimahoracl ...
8	2	radioeme, lavozdemaipu
9	6	eldia_cl, diariolabrador, somosmelipilla, portaldemeli, elcomunicadorcl, diarioelcentro
10	2	chilemosaico, proclamacion
11	2	laperladelimari, diarioovallehoy
12	2	prensatuciudad, rengonotas
13	2	mundoacuicola, aquasocial
14	2	radiocorazonfm,radiopudahuel

The cluster with ID 0 corresponds to un-grouped media outlets.
