# Supplementary material for: Power structure in Chilean news media
Source: PLoS One. 2018 Jun 6;13(6):e0197150. doi: 10.1371/journal.pone.0197150 (PMC5991387; doi:10.1371/journal.pone.0197150)
Supplement: S2 Table — The cluster with ID 15 corresponds to un-grouped media outlets. Entities owning over 10% of the outlets in a community are listed next to it. (PDF) [file pone.0197150.s002.pdf]

S2 Table. Ownership properties for Vocabulary-based clusters for the *ds16* dataset.

Com. ID	Size	Main owners	Owner %	Unknown owner %
0	3	el conquistador fm	33.33	66.67
1	11	el mercurio<br>asesorias e inversiones comunidades ciudadanas	18.18<br>18.18	54.55
2	9	sociedad radiodifusora y periodistica del maule<br>radiodifusora paloma<br>portales regionales<br>grupo diarios en red<br>asesorias e inversiones comunidades ciudadanas<br>luis verdejo vega<br>empresa periodistica curico<br>sociedad radiodifusora cheis	11.11<br>11.11<br>11.11<br>11.11<br>11.11<br>11.11<br>11.11<br>11.11	11.11
3	16	el mercurio	100.00	0.00
4	41	copesa<br>grupo metro internacional<br>editorial televisa chile	21.95<br>12.20<br>12.20	0.00
5	12	grupo diarios en red	100.00	0.00
6	28	el mercurio<br>asesorias e inversiones comunidades ciudadanas	14.29<br>25.00	21.43
7	6	medios de consorcio periodistico el epicentro<br>el mercurio<br>corporacion de television de la pontificia universidad catolica de valparaiso<br>comunicaciones pacifico<br>asesorias e inversiones comunidades ciudadanas	16.67<br>33.33<br>16.67<br>16.67<br>16.67	0.00
8	19	el mercurio<br>universidad de concepcion	21.05<br>10.53	21.05
9	38	copesa	13.16	10.53
10	4	sociedad periodistica e impresora el labrador<br>portal de melipilla	25.00<br>25.00	50.00
11	36	inversiones canal 13<br>grupo prisa	11.11<br>13.89	13.89
12	17	–	–	29.41
13	10	el mercurio	40.00	40.00
14	12	red de diarios comunales	41.67	8.33
15	66	–	–	24.24
16	8	el mercurio<br>gestion y comunicaciones san lorenzo<br>alberto bichara<br>enfoque digital<br>asesorias e inversiones comunidades ciudadanas	12.50<br>12.50<br>12.50<br>12.50<br>12.50	37.50
17	4	fundacion democracia y desarrollo<br>el democrata<br>sociedad periodistica el libero	25.00<br>25.00<br>25.00	25.00

The cluster with ID 15 corresponds to un-grouped media outlets. Entities owning over 10% of the outlets in a community are listed next to it.
