# Supplementary material for: Power structure in Chilean news media
Source: PLoS One. 2018 Jun 6;13(6):e0197150. doi: 10.1371/journal.pone.0197150 (PMC5991387; doi:10.1371/journal.pone.0197150)
Supplement: S3 Table — The cluster with ID 7 corresponds to un-grouped media outlets. Entities owning over 10% of the outlets in a community are listed next to it. (PDF) [file pone.0197150.s003.pdf]

S3 Table. Ownership properties for Vocabulary clusters for the *ds15* dataset.

Com. ID	Size	Main owners	Owner %	Unknown owner %
0	14	el mercurio	57.14	7.14
		estado de chile	14.29	
1	4	sociedad informativa regional	25.00	50.00
		asesorias e inversiones comunidades ciudadanas	25.00	
2	9	el mercurio	11.11	11.11
		sociedad periodistica de aysen	11.11	
		sociedad periodistica el ciudadano	11.11	
		patagonica publicaciones	11.11	
		la plaza	11.11	
		ediciones y publicaciones bobby	11.11	
		sociedad editora y periodistica la verdad	11.11	
		empresa de publicaciones la prensa austral	11.11	
3	2	el mercurio	50.00	0.00
		universidad de concepcion	50.00	
4	3	el mercurio	33.33	33.33
		asesorias e inversiones comunidades ciudadanas	33.33	
5	2	el mercurio	50.00	0.00
		asesorias e inversiones comunidades ciudadanas	50.00	
6	7	el mercurio	14.29	14.29
		grupo mosciatti	14.29	
		universidad de concepcion	14.29	
		asesorias e inversiones comunidades ciudadanas	14.29	
		sociedad nacional de agricultura	14.29	
		empresa periodistica curico	14.29	
7	11	–	–	0.00
8	3	el mercurio	33.33	33.33
		asesorias e inversiones comunidades ciudadanas	33.33	
9	3	el mercurio	33.33	33.33
		asesorias e inversiones comunidades ciudadanas	33.33	
10	6	asesorias e inversiones comunidades ciudadanas	100.00	0.00
11	3	el mercurio	100.00	0.00
12	4	el mercurio	25.00	25.00
		empresa periodistica el observador	25.00	
		marcelo jara olivares	25.00	
13	8	sociedad periodistica e impresora el labrador	12.50	12.50
		copesa	12.50	
		asesorias e inversiones comunidades ciudadanas	12.50	
		carabineros de chile	12.50	
		editora el centro empresa periodistica	12.50	
		antonio puga	25.00	

The cluster with ID 7 corresponds to un-grouped media outlets. Entities owning over 10% of the outlets in a community are listed next to it.
