# Supplementary material for: Power structure in Chilean news media
Source: PLoS One. 2018 Jun 6;13(6):e0197150. doi: 10.1371/journal.pone.0197150 (PMC5991387; doi:10.1371/journal.pone.0197150)
Supplement: S4 Table — The cluster with ID 0 corresponds to un-grouped media. (PDF) [file pone.0197150.s004.pdf]

**S4 Table. News outlets for Topic keyword-based communities for the *ds16* dataset.**

| Com. ID | Size | Outlets |
|---|---|---|
| 0 | 208 | elvicunense, canal_13c, diariodeaysen, diarioelcomunal, sabrosia, bolido_com, 40chileoficial ... |
| 1 | 59 | cooperativa, nacioncl, t13, chilebcl, radionuble, eldia_cl, gamba_cl, emol, eldesconcierto, lacuarta ... |
| 2 | 14 | austral_osorno, austral_losrios, ellanquihue, estrellaconce, clave9cl, diarioatacama, estrelladearica ... |
| 3 | 4 | radiovalparaiso, radio_festival, elepicentro, ucvradio |
| 4 | 16 | uchileradio, elrepuertero, laopinon, el_amaule, elvacanudo, elnaveghable, elmagallanews, elobservatodo ... |
| 5 | 25 | soysanantonio, soytome, soyantofagasta, laestrellavalpo, soytemuco, laestrellaiqq, soycopiapo ... |
| 6 | 13 | redarica, diariosenred, red_coquimbo, redantofagasta, redaraucania, redmaule, redbiobio, redlosrios ... |
| 7 | 2 | putaendoinforma, aconcaguanews |

The cluster with ID 0 corresponds to un-grouped media outlets.
