# Supplementary material for: Power structure in Chilean news media
Source: PLoS One. 2018 Jun 6;13(6):e0197150. doi: 10.1371/journal.pone.0197150 (PMC5991387; doi:10.1371/journal.pone.0197150)
Supplement: S5 Table — The cluster with ID 1 corresponds to un-grouped media outlets. Entities owning over 10% of the outlets in a community are listed next to it. (PDF) [file pone.0197150.s005.pdf]

S5 Table. Ownership properties for Topic keyword-based clusters for the *ds16* dataset.

Com. ID	Size	Main owners	Owner %	Unknown owner %
0	13	grupo diarios en red	100.00	0.00
1	158	–	–	27.85
2	1	el mercurio	100.00	0.00
3	15	asesorias e inversiones comunidades ciudadanas	100.00	0.00
4	12	el mercurio	100.00	0.00
5	13	–	–	0.00
6	5	medios de consorcio periodistico el epicentro	20.00	0.00
		el mercurio	40.00	
		corporacion de television de la pontificia universidad catolica de valparaiso	20.00	
		comunicaciones pacifico	20.00	
7	30	copesa	13.33	3.33
		grupo prisa	20.00	
8	3	el mercurio	100.00	0.00
9	5	sociedad de comunicaciones el trabajo	20.00	20.00
		marcelo jara olivares	20.00	
		sociedad radio aconcagua	20.00	
		patricio gallardo montenegro	20.00	
10	23	copesa	13.04	17.39
11	34	–	–	20.59
12	16	el mercurio	100.00	0.00

The cluster with ID 1 corresponds to un-grouped media outlets. Entities owning over 10% of the outlets in a community are listed next to it.
