# Supplementary material for: Power structure in Chilean news media
Source: PLoS One. 2018 Jun 6;13(6):e0197150. doi: 10.1371/journal.pone.0197150 (PMC5991387; doi:10.1371/journal.pone.0197150)
Supplement: S6 Table — The cluster with ID 1 corresponds to un-grouped media outlets. Entities owning over 10% of the outlets in a community are listed next to it. (PDF) [file pone.0197150.s006.pdf]

S6 Table. Ownership properties for Topic keyword-based clusters for the *ds15* dataset.

Com. ID	Size	Main owners	Owner %	Unknown owner %
0	9	el mercurio	11.11	0.00
		estado de chile	11.11	
		grupo mosciatti	11.11	
		copesa	11.11	
		terra networks chile	11.11	
		fundacion para las comunicaciones sociales	11.11	
		comunicaciones lanet	11.11	
		salvador schwartzmann	11.11	
		inversiones canal 13	11.11	
1	28	–	–	32.14
2	5	el mercurio	100.00	0.00
3	7	el mercurio	100.00	0.00
4	12	asesorias e inversiones comunidades ciudadanas	100.00	0.00
5	3	el mercurio	100.00	0.00
6	2	asesorias e inversiones comunidades ciudadanas	100.00	0.00
7	7	el mercurio	14.29	14.29
		cnn chile	14.29	
		sociedad periodistica el ciudadano	14.29	
		ediciones y publicaciones bobby	14.29	
		universidad de concepcion	14.29	
		la plaza	14.29	
8	2	el mercurio	100.00	0.00
9	4	copesa	25.00	0.00
		grupo bethia	25.00	
		grupo metro internacional	25.00	
		grupo prisa	25.00	

The cluster with ID 1 corresponds to un-grouped media outlets. Entities owning over 10% of the outlets in a community are listed next to it.
