# Supplementary material for: Power structure in Chilean news media
Source: PLoS One. 2018 Jun 6;13(6):e0197150. doi: 10.1371/journal.pone.0197150 (PMC5991387; doi:10.1371/journal.pone.0197150)
Supplement: S7 Table — The cluster with ID 0 corresponds to un-grouped media outlets. (PDF) [file pone.0197150.s007.pdf]

S7 Table. **News outlets for Topic minhash-based communities for the *ds16* dataset.**

Com. ID	Size	Outlets
0	10	ahoranoticiasan, carabdechile, chilebcl, diarioenaccion, futurafmoficial, hoyxhoycl, la_segunda ...
1	109	24horastvn, 40chileoficial, adnradiochile, antofacity_com, armoniaonline, biobio, biobiodeportivo ...
2	43	33temuco, austral_losrios, austral_osorno, australtemuco, cronicachillan, diarioatacama, diarioelhuemul ...
3	130	acciondeongs, aconcaguanews, aconcaguaradio, agriculturafm, alfaomegacurico, americaeconomia, antofagastatv ...
4	2	ancoafm, canal5linares
5	2	aquasocial, mundoacuicola
6	9	betazeta, bolido_com, chw_net, fayerwayer, ferplei, niubie_com, sabrosia, veoverde, wayerless
7	36	carta_abierta, cnnchile, concordia_arica, diario_eha, diariolabrador, el_amaule, el_provincial ...
8	6	chiloealdia, elinsular1, estrellachiloe, lacuarta, queilencl, radioquellon
9	14	diariosenred, elliberocl, red_coquimbo, red_ohiggins, redantofagasta, redaraucania, redarica, redatacama ...
10	2	lavozdemaipu, radioeme
11	2	primordialfm, ultimahoracl

The cluster with ID 0 corresponds to un-grouped media outlets.
