# Supplementary material for: Power structure in Chilean news media
Source: PLoS One. 2018 Jun 6;13(6):e0197150. doi: 10.1371/journal.pone.0197150 (PMC5991387; doi:10.1371/journal.pone.0197150)
Supplement: S8 Table — The cluster with ID 0 corresponds to un-grouped media outlets. Entities owning over 10% of the outlets in a community are listed next to it. (PDF) [file pone.0197150.s008.pdf]

S8 Table. Ownership properties for Topic minhash-based clusters for the *ds15* dataset.

Com. ID	Size	Main owners	Owner %	Unknown owner %
0	31	–	–	22.58
1	14	el mercurio	100.00	0.00
2	11	asesorias e inversiones comunidades ciudadanas	100.00	0.00
3	1	el mercurio	100.00	0.00
4	1	grupo mosciatti	100.00	0.00
5	2	copesa	50.00	50.00
6	3	alberto bichara	33.33	0.00
		universidad de concepcion	33.33	
		empresa de publicaciones la prensa austral	33.33	
7	3	sociedad periodistica e impresora el labrador	33.33	0.00
		grupo metro internacional	33.33	
		grupo bethia	33.33	
8	4	marcelo jara olivares	25.00	0.00
		terra networks chile	25.00	
		asesorias e inversiones comunidades ciudadanas	50.00	
9	2	antonio puga	100.00	0.00
10	2	estado de chile	100.00	0.00
11	4	sociedad informativa regional	25.00	50.00
		asesorias e inversiones comunidades ciudadanas	25.00	
12	1	fundacion para las comunicaciones sociales	100.00	0.00
13	2	el mercurio	100.00	0.00

The cluster with ID 0 corresponds to un-grouped media outlets. Entities owning over 10% of the outlets in a community are listed next to it.
